# The dataset of methylglyoxal activating p38 and p44/42 pathway in osteoclast

**DOI:** 10.1016/j.dib.2019.104500

**Published:** 2019-09-17

**Authors:** Kwan Ming Lee, Cheuk Yan Lee, Ge Zhang, Aiping Lyu, Kevin Kin Man Yue

**Affiliations:** a School of Chinese Medicine, Hong Kong Baptist University, Hong Kong, China; b Institute for Advancing Translational Medicine in Bone & Joint Diseases, Hong Kong Baptist University, Hong Kong, China

**Keywords:** Methylglyoxal, Diabetes, Osteoporosis, Osteoclast, Pathways

## Abstract

Diabetes mellitus (DM) is a kind of chronic metabolic disease that could be characterized by uncontrollable high blood glucose (hyperglycemia) over a prolonged period and diverse complications in various organs. These complications include activation of stress responses in bone such as oxidative stress and inflammation, which have been implicated in various bone diseases, including osteoporosis. Non-enzymatic glycation of proteins form and accumulate in patients under hyperglycemia condition. Methylglyoxal (MG) is a reactive advanced glycation end-product precursor.

Abnormal high concentration of MG was in serum of diabetic patients. It was proven that MG induces various stress responses. This indicates that it might possibly the key metabolite leading to diabetes-associated bone loss. In this data report, using cell models, the underlying mechanism of methylglyoxal on osteoclast that may lead to bone loss was investigated.

In cell cultures, RAW264.7, Macrophages, was treated with methylglyoxal and gene expressions of osteoclast bone biomarkers were investigated. Furthermore, the inhibitions of p38 and p44/42 activities were employed to investigate the osteoclast biomarkers CTSK, OSCAR, and TRACP5 gene expressions.

These data implied that MG activated the p38 and p44/42, which was reported to regulate proliferation and differentiation of osteoclast. However, the decreasing MAPK though siRNA knockdown did not change expression of those target markers, TRACP5, OSCAR, and CTSK, in mRNA level. The effects of MG to other osteoclast markers through p38 and p44/42 would be worth to be investigated.

For more insight please see Methylglyoxal Activates Osteoclasts through JNK Pathway leading to Osteoporosis.

**Table:** Specifications Table

Subject	Biology
Specific subject area	Endocrinology, Diabetes and Metabolism
Type of data	Chart Figure
How data were acquired	Real time-PCR, Western blot analysis
Data format	Raw Analyzed
Parameters for data collection	*In vitro,* Under Methylglyoxal treatment
Description of data collection	Macrophages, cell line RAW264.7, were treated with MG and collected at different time intervals after the MG treatment. In the knockdown study, cells were treated with 10 nM of MAPK3 siRNA or MAPK14 siRNA (Invitrogen) and siRNA (Invitrogen) used as the negative control in Opti-MEM reduced serum culture medium for 24 h before MG treatment. mRNA and Proteins of samples were then collected for further study and analysis.
Data source location	Institution: School of Chinese Medicine, Hong Kong Baptist University City/Town/Region: Hong Kong Country: China Latitude and longitude (and GPS coordinates) for collected samples/data: 22.335550, 114.182349]
Data accessibility	Na
Related research article	Authors: Kevin Yue, Kwan Ming Lee, Cheuk Yan Lee, Ge Zhang, Aiping Lu Title: Methylglyoxal activates osteoclasts through JNK pathway leading to osteoporosis Journal: Chemico-Biological Interactions https://doi.org/10.1016/j.cbi.2019.05.026.

**Table:** 

**Value of the Data** • The inhibition of p38 activities were employed to investigate the osteoclast biomarkers CTSK, OSCAR, and TRACP5 gene expressions. • Using respective siRNA, the knockdown of p38 and p44/42 did not show the corresponding down-regulation in mRNA expression of TRACP5, OSCAR, and CTSK. • After 1 h MG treatment, phosphorylation of p38, and p44/42 both increased and reached a maximum • The analysis contributed to the study of the role of methylglyoxal causing bone loss in osteoclast cells and offered an opportunity to find the cause of diabetic bone loss. • Further studies in pathways other than p38 and p44/42, which may case bone loss by methylglyoxal, are desirables.

## 1 Data

**Fig. 1:**
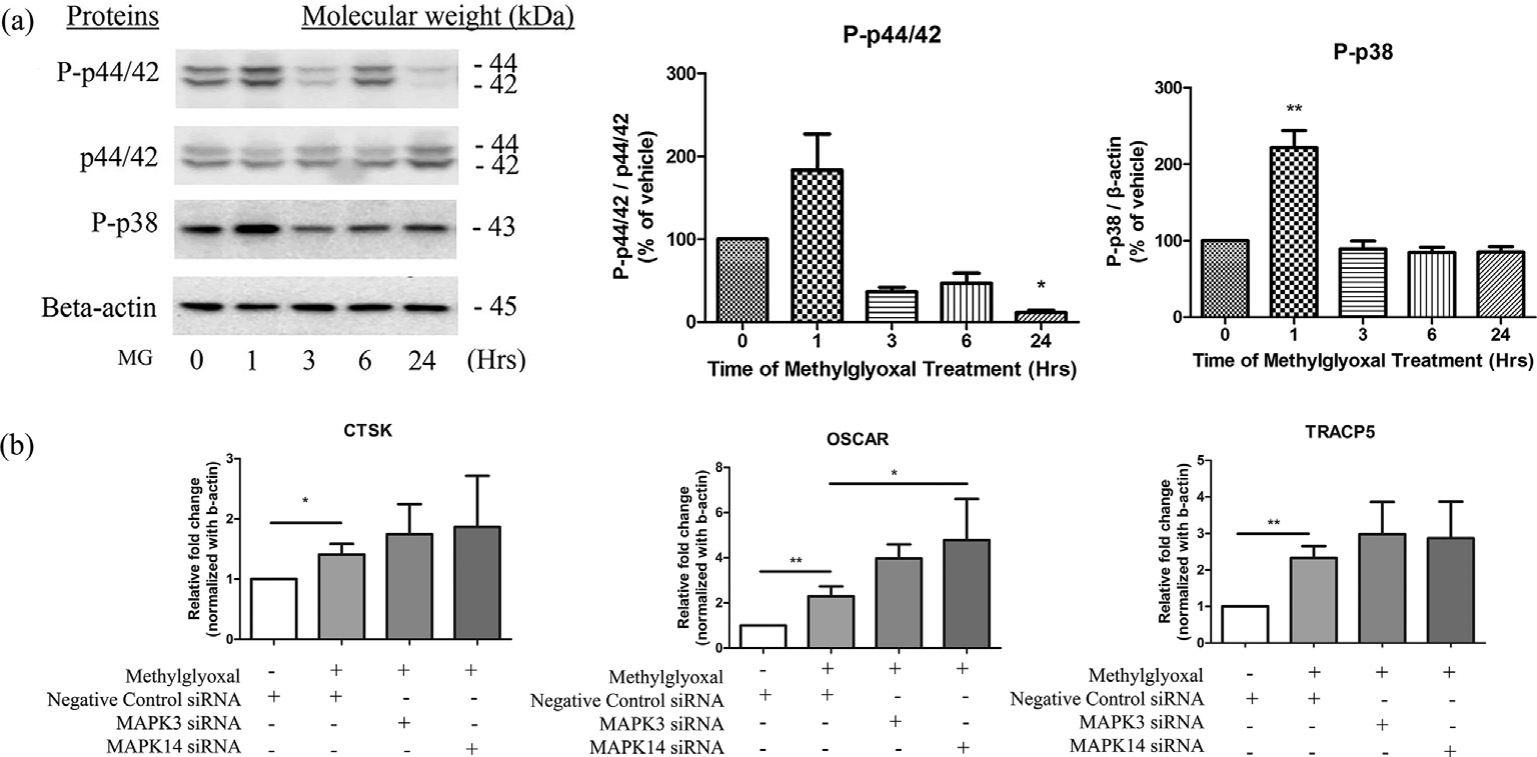
The band densities quantification of Western Blotting was shown in the graph. In Fig. 1(a), Representative blots of p-p38, p-p44/42, p44/42, and beta-actin of the cells after exposing to MG (400 μM) after the treatment time intervals between 1 h and 24 h. Phosphorylation of p38 and p44/42 were induced by MG in RAW264.7 cells. Endogenous p44/42 and beat-actin were used as the internal control. Data shown was corresponding to 5 repeated experiments.

The band densities quantification of Western Blotting was shown in the graph below. In Fig. 1a, Representative blots of p-p38, p-p44/42, p44/42, and beta-actin of the cells after exposing to MG (400 μM) after the treatment time intervals between 1 h and 24 h. Phosphorylation of p38 and p44/42 were induced by MG in RAW264.7 cells. Endogenous p44/42 and beat-actin were used as the internal control. Data shown was corresponding to 5 repeated experiments.

In Fig. 1b, the mRNA expression of osteoclast biomarkers: TRACP5, OSCAR, and CTSK still increased in MG group and the effects of MG did not countered by the transfection. Before 400 μM MG treatment, RAW264.7 cells underwent transfection under treatment of 10 nM siRNA for 24 h. The cells were collected after 24 h MG treatment. However, the mRNA expression of the osteoclast bone biomarkers persisted to increase under p38 and p44/42 inhibition in MG-treated macrophages. Beta-actin acted as mRNA internal control. Data shown was corresponding to 5 repeated experiments.

These data implied that MG activated the p38 and p44/42, which was reported to regulate proliferation and differentiation of osteoclast. However, the decreasing MAPK though siRNA knockdown did not change expression of those target markers, TRACP5, OSCAR, and CTSK, in mRNA level. The effects of MG to other osteoclast markers through p38 and p44/42 would be worth to be investigated.

## 2 Experimental design, materials, and methods

### 2.1 Chemicals

Methylglyoxal was purchased from Sigma (St. Louis, MO, USA). *Anti*-β-actin antibody was purchased from Sigma (St. Louis, MO, USA). Lipofectamine® 2000 Transfection Reagent and siRNA were both obtained from Invitrogen (Carlsbad, CA, USA). Remaining antibodies that were used in this study were ordered from Cell Signaling Technology (Beverly, MA, USA). Other chemicals of reagent-grade quality were obtained from Sigma (St. Louis, MO, USA).

### 2.2 RAW264.7 treatments and cell cultures

In the study of the cell model, a transfect-able macrophage cell line with the capacity to form osteoclast-like cells, RAW264.7 macrophages (ATCC, TIB-71), was employed. RAW264.7 were cultured in DMEM supplemented with 10% fetal bovine serum, and 1% antibiotic-antimycotic (Invitrogen, Carlsbad, CA, USA) at a humidified atmosphere with 5% CO_2_ at 37 °C. Cells were seeded on 96 and 6 well plates respectively for experimental use. To conduct MTT assay, 24 hours after cell seeding on 96 well plates, cells were treated with MG or vehicle control for 24 hours. For the 6 wells plates, cells were treated with MG and collected at different time intervals (1 h, 3 h, 6 h and 24 h) after the MG treatment. After that, proteins and mRNA of cells were collected for further studies. To conduct experiments of inhibiting p38 and p44/42, cells were treated with 10 nM p38 (MAPK14) and p44/42 (MAPK3) siRNA in Opti-MEM reduced serum culture medium respectively for 24 h before MG treatment. For the negative control, siRNA was added onto cells in Opti-MEM reduced serum culture medium for 24 h before MG treatment. After transfection, cells were treated in MG (400 μM) for 24 h. After that, mRNA of cells was extracted for further studies.

### 2.3 Western Blotting analysis

Proteins from the cells were extracted with radioimmunoprecipitation assay buffer (RIPA) with protease inhibitors containing Complete Mini™ protease inhibitor cocktail (Roche), 1 mM PMSF, and 1 mM Na_2_VO_4_. Protein samples were then separated by SDS-PAGE gel and transferred to the PVDF Western Blotting membrane. The membranes were later probed with respective primary antibodies overnight at 4 °C, and then respective HRP-conjugated secondary antibodies for 1 h. The membranes were incubated with ECL reagents and stained protein bands were exposed and captured using Bio-Rad ChemiDoc Touch Imaging System. The intensities of the protein bands were quantified using Image J, an image-processing program.

### 2.4 mRNA extraction and real-time PCR

The endogenous mRNA in cell samples was extracted, using Trizol (Invitrogen). The mRNA was then converted into complementary DNA (cDNA) under reverse transcription. Expressions of the osteoclast biomarkers were assessed by real-time PCR with respective primers:

1) Mouse CTSK (F: CCCTTAGTCTTCCGCTCACA, R: TCCTCCGGAGACAGAGCAAA);
2) Mouse OSCAR (F: GTCCTGTCGCTGATACTCCAG, R: CCCAGTCTGTCTTGCGGTAG);
3) Mouse TRACP5 (F: GCAGCTCCCTAGAAGATGGAT, R: AACACGTCCTCAAAGGTCTCC);
4) Mouse β-Actin (F: TAGGCGGACTGTTACTGAGC, R: TGCTCCAACCAACTGCTGTC).

### 2.5 Statistical test

Results were shown as means ± standard deviation (SD). The data were processed using Student's t-test and one-way analysis of variance (ANOVA) followed by Tukey's range test. While P < 0.05 was considered statistically significant (*p < 0.05 in comparison to the control. **p < 0.01 in comparison to the control).

